# Biofilm formation and toxin production provide a fitness advantage in mixed colonies of environmental yeast isolates

**DOI:** 10.1002/ece3.4082

**Published:** 2018-04-27

**Authors:** Bernadette M. Deschaine, Angela R. Heysel, B. Adam Lenhart, Helen A. Murphy

**Affiliations:** ^1^ Department of Biology The College of William and Mary Williamsburg Virginia

**Keywords:** biofilm, colony morphology, fluffy colony, K2, *Saccharomyces cerevisiae*

## Abstract

Microbes can engage in social interactions ranging from cooperation to warfare. Biofilms are structured, cooperative microbial communities. Like all cooperative communities, they are susceptible to invasion by selfish individuals who benefit without contributing. However, biofilms are pervasive and ancient, representing the first fossilized life. One hypothesis for the stability of biofilms is spatial structure: Segregated patches of related cooperative cells are able to outcompete unrelated cells. These dynamics have been explored computationally and in bacteria; however, their relevance to eukaryotic microbes remains an open question. The complexity of eukaryotic cell signaling and communication suggests the possibility of different social dynamics. Using the tractable model yeast, *Saccharomyces cerevisiae*, which can form biofilms, we investigate the interactions of environmental isolates with different social phenotypes. We find that biofilm strains spatially exclude nonbiofilm strains and that biofilm spatial structure confers a consistent and robust fitness advantage in direct competition. Furthermore, biofilms may protect against killer toxin, a warfare phenotype. During biofilm formation, cells are susceptible to toxin from nearby competitors; however, increased spatial use may provide an escape from toxin producers. Our results suggest that yeast biofilms represent a competitive strategy and that principles elucidated for the evolution and stability of bacterial biofilms may apply to more complex eukaryotes.

## 1 INTRODUCTION

Social interactions between microbes, both within and between species, are abundant and extremely important. Such interactions can include cooperation, competition, synchronization, and even chemical warfare (West, Diggle, Buckling, Gardner, & Griffin, 2007). Biofilms are cooperative microbial communities composed of one or multiple species, anchored to a surface, and protected from environmental hazards by a secreted extracellular matrix (Lee et al., 2014; O'Toole, Kaplan, & Kolter, 2000). They are found throughout the natural and man‐made environment, wherever microbes are found—from hulls on ships (Little, Lee, & Ray, 2008) to dental surfaces (Kolenbrander, 2000). Biofilms also protect microbes from antibiotics and can therefore cause persistent infections (Costerton, Stewart, & Greenberg, 1999). The oldest fossils on earth are microbial mats; thus, it appears that there have been biofilms since microbes first evolved (Nutman, Bennett, Friend, Van Kranendonk, & Chivas, 2016).

Biofilms require individuals to produce goods, such as components of the extracellular matrix, that can be used by all members. Like all cooperative communities, they are susceptible to “cheaters” who do not produce the public goods, yet benefit from them (Brockhurst, Buckling, & Gardner, 2007; Crespi, 2001; Rainey & Rainey, 2003; Smukalla et al., 2008; West, Griffin, Gardner, & Diggle, 2006). Despite their vulnerability to individual cheaters, biofilms are ubiquitous and stable. The leading hypothesis for the stability of biofilm communities is the spatial structure: Competition, cooperation, and passive processes like clonal growth can generate patches of related cooperative cells able to outcompete unrelated cells (e.g., (Anderson, Garcia, & Cotter, 2014; van Gestel, Weissing, Kuipers, & Kovacs, 2014; Hallatschek, Hersen, Ramanathan, & Nelson, 2007; Millet et al., 2014; Momeni, Brileya, Fields, & Shou, 2013; Müller, Neugeboren, Nelson, & Murray, 2014; Nadell & Bassler, 2011; Nadell, Foster, & Xavier, 2010; Van Dyken, Müller, Mack, & Desai, 2013; Xavier & Foster, 2007), recently reviewed in detail in ref. (Nadell, Drescher, & Foster, 2016)). Aside from acting as a public good, the production of substances that facilitate cell‐to‐cell and cell‐to‐surface adherence can be a competitive cooperative strategy that allows lineages increased access to space and nutrients (Garcia, Doulcier, & De Monte, 2015; Irie et al., 2017; Kim, Racimo, Schluter, Levy, & Foster, 2014; Xavier & Foster, 2007) and can even work to exclude nonproducers from the community (Schluter, Nadell, Bassler, & Foster, 2015). Recent work in the bacterium *Pseudomonas aeruginosa* demonstrated that when multiple strains were grown together, biofilm formation increased, and single strains often dominated the competition (Oliveira et al., 2015).

Another type of competitive strategy in microbial communities is warfare, which takes the form of microbial toxins and antibiotics (Riley & Wertz, 2002; Schmitt & Breinig, 2006). Under certain conditions, warfare‐producing and sensitive lineages can coexist within an expanding spatially structured community (Abrudan et al., 2015; Bucci, Nadell, & Xavier, 2011; Gardner & West, 2004; Tait & Sutherland, 2002; Weber, Poxleitner, Hebisch, Frey, & Opitz, 2014). It has also recently been demonstrated that in a dense, well‐mixed community, a warfare phenotype can generate spatial segregation of producing and sensitive lineages (McNally et al., 2017). The interaction between microbes producing warfare phenotypes and microbes producing biofilms is not yet entirely clear. The same study that investigated multistrain *P. aeruginosa* communities (Oliveira et al., 2015) also found that the production of antibiotics by competitors increased biofilm formation. This suggests that biofilms may serve to protect from warfare phenotypes.

Most research on microbial social evolution has been conducted in bacterial systems (Nadell et al., 2016; West & Cooper, 2016; West et al., 2006, 2007). However, the complexity of eukaryotic cell structures, communication, and gene regulation, and the potential differences between bacterial and fungal biofilms (Blankenship & Mitchell, 2006) leave open the possibility that the social dynamics may be quite different in eukaryotic microbes. Furthermore, the relevance of fungal biofilms to public health (Nobile & Johnson, 2015) suggests that understanding the social and evolutionary dynamics within a fungal model is of increasing importance. Pathogenic species of the yeast genus *Candida* can form drug‐resistant biofilms on medical devices—most notably catheters, heart implants, and joint replacements—and are a major source of hospital‐acquired infections (Chandra et al., 2001; Douglas, 2003).

### 1.1 *Saccharomyces cerevisiae* social phenotypes

Cells of the model yeast, *Saccharomyces cerevisiae*, can adhere to each other and various surfaces, forming biofilm mats and colonies (Kuthan et al., 2003; Reynolds & Fink, 2001; Verstrepen & Klis, 2006), and can also engage in warfare through toxins (Schmitt & Breinig, 2006). These social phenotypes are common in environmental isolates (Granek & Magwene, 2010; Hope & Dunham, 2014; Pieczynska, de Visser, & Korona, 2013), making *S. cerevisiae* an ideal model to study fungal biofilms (Bojsen, Andersen, & Regenberg, 2012) and investigate questions related to eukaryotic sociomicrobiology. Furthermore, a study investigating a potentially cooperative phenotype in liquid, flocculation, showed that formation of flocs provided protection against environmental stressors and was regulated by a “greenbeard” locus (Smukalla et al., 2008), thus suggesting the potential for cooperation in spatially structured communities as well.

A spatially explicit cooperative yeast phenotype is complex colony morphology (“fluffy”), which resembles the wrinkly colonies of the bacterial biofilm models *P. aeruginosa* and *Bacillus subtilis*, and has all the hallmarks of fungal biofilms (Blankenship & Mitchell, 2006): An extracellular matrix facilitating nutrient flow and water retention (Kuthan et al., 2003; Štovíček, Váchová, Kuthan, & Palková, 2010); expression of drug efflux pumps; and velcro‐like structures attaching cells to one another (Váchová et al., 2011) encoded by an adhesin gene, *FLO11* (Kraushaar et al., 2015). When grown as single‐strain colonies (Tan et al., 2013) or mats (Regenberg, Hanghøj, Andersen, & Boomsma, 2016), strains forming biofilms have been shown to spread and occupy space more quickly than non‐biofilm‐forming (smooth) strains; however, smooth colonies have a greater cell density (Štovíček et al., 2010). Thus, cell counts, rather than colony size, should be used to test the fitness effects of biofilm formation. While simple smooth *S. cerevisiae* colonies have been used to explore spatially expanding mixed populations (Korolev et al., 2012; Momeni et al., 2013; Müller et al., 2014; Van Dyken et al., 2013), and one study has generated mixed *FLO11* and *flo11* colonies from a single laboratory background (Chen et al., 2014), to our knowledge, the evolutionary dynamics of multistrain biofilm communities have not been explored.

Killer toxins represent a yeast warfare phenotype and a natural antifungal. They are secreted proteins that function in interstrain competition: Secreting cells are protected, while nearby sensitive cells are killed (Schmitt & Breinig, 2006). Killer toxins are encoded by cytoplasmically inherited double‐stranded RNA (dsRNA) viruses; they replicate with the aid of dsRNA helper viruses (Schmitt & Breinig, 2006). Toxins occur widely in natural populations of *Saccharomyces* yeasts, with toxin production detected in ~10% of strains surveyed from publicly available collections (Pieczynska et al., 2013). The research presented here focuses on K2 (Wingfield, van der Meer, Pretorious, & Vvan Vuuren, 1990), the killer toxin most commonly found in vineyard ecosystems (Pieczynska et al., 2013). It acts quickly to induce membrane permeability and reduce intracellular ATP levels in sensitive cells, but the details of its mode of action remain unknown (Orentaite, Poranen, Oksanen, Daugelavicius, & Bamford, 2016). It remains unstudied whether biofilms protect yeast against killer toxin, or whether killer toxin is able to penetrate biofilms.

### 1.2 This study

We sought to test the generality of the predictions of microbial social evolution theory for spatially structured communities that have been demonstrated in silico and in bacteria: that biofilm formation provides a strong fitness benefit and that biofilms are a competitive strategy used to obtain resources and exclude other strains (Garcia et al., 2015; Kim et al., 2014; Schluter et al., 2015; Xavier & Foster, 2007). If these results hold true in more complex eukaryotes, they may represent universal principles underlying the stability of biofilms. We first explored whether clonal growth and spatial structure provide fitness benefits in yeast. Based on computational and experimental work with bacterial species, we hypothesized that biofilm formers would outcompete nonbiofilm formers for both space and resources. Next, we tested whether biofilm production protected cells from the warfare phenotype killer toxin, an area of microbial social evolution with less background theoretical and experimental research. We predicted that biofilm formation would protect clonal lineages from this antifungal warfare phenotype, as many bacterial biofilms provide protection against antibiotics.

Our experiments used environmental isolates in order to understand how social phenotypes interact during ecological competition. *S. cerevisiae* is found in a variety of ecological niches (Cromie et al., 2013; Liti et al., 2009; Schacherer, Shapiro, Ruderfer, & Kruglyak, 2009; Strope et al., 2015), and insects have been shown to transport the yeast and to increase outcrossing rates (Goddard, Anfang, Tang, Gardner, & Jun, 2009; Reuter, Bell, & Greig, 2007; Stefanini et al., 2012, 2016). This suggests that different genetic backgrounds likely interact in nature and may directly compete with one another. We therefore directly and indirectly competed isolates of *S. cerevisiae* in spatially structured communities, using cell counts to determine the fitness effects of biofilm formation and toxin production. Our experimental results demonstrated a consistent and robust fitness benefit to yeast biofilm formation in direct competition, thus supporting the idea that biofilms may be a competitive phenotype for genetic lineages. Furthermore, toxin production was effective against biofilm‐forming strains, although we speculate that spatial use may provide a way to escape from toxin‐producing competitors.

## 2 MATERIALS AND METHODS

### 2.1 Strains

Diploid *S. cerevisiae* strains from publicly available (Liti et al., 2009; Strope et al., 2015) and personal collections were screened using the classification system of Granek and Magwene (2010) (Table S1). Smooth strains and biofilm‐forming strains with distinct colony morphologies and from different ecological niches were identified. Two biofilm strains—YJM311 (clinical) (McCusker, Clemons, Stevens, & Davis, 1994), YJM224 (distillery yeast)—and three smooth strains—YJM981 (clinical) (McCullough, Clemons, Farina, McCusker, & Stevens, 1998), SK1 (lab/soil) (Liti et al., 2009), YPS681 (woodland) (Sniegowski, Dombrowski, & Fingerman, 2002)—were selected for fitness assays.

### 2.2 Incorporating fluorescence and antibiotic markers

The diploid isolates were transformed using a lithium acetate procedure (Gietz & Woods, 2002) with a cassette that targeted the terminal region of the highly expressed *PGK1* gene (Figure S2) and contained: (1) either mCherry or GFP, and (2) antibiotic resistance through KanMX (Wach, Brachat, Pohlmann, & Philippsen, 1994), NatMX or HphMX (Goldstein & McCusker, 1999). Plasmids pFA6a‐GFP‐KanMX6 (Longtine et al., 1998) and pBS34‐mCherry‐KanMX6 (Hailey, Davis, & Muller, 2002) were digested with NotI and used as template for PCR (Yeast Resource Center, University of Washington). For some strains, the antibiotic resistance in fluorescently labeled yeast was subsequently switched via transformation with NatMX or HphMX (Table S1). All polymerase chain reactions were performed with iProof polymerase (Bio‐Rad) using the manufacturer's recommendations for cycling conditions using the primers listed in Table S2; DMSO was added to 3% to reaction mixtures. Strains with killer toxin virus K2 (29‐06) (Pieczynska et al., 2013) (generously provided by D. Wloch‐Salamon) were used for assays with toxin activity. K2 is active in the acidic pH range of 2.5–5.0 at temperatures between 20 and 25°C (Lukša, Serva, & Servienė, 2016).

### 2.3 Media

Strains were grown in YPD (1% yeast extract, 2% peptone, 2% dextrose) or low dextrose (LD) YPD (0.1% dextrose); solid media contained 2% agar. When appropriate, media was supplemented with 150 μg/ml G418, 75 μg/ml CloNat, or 300 μg/ml hygromycin B. Toxin assays were performed on 1.5% agar YPD and LD‐YPD plates supplemented with citric acid to adjust the pH to 4.5 (~3 mg) (Lukša et al., 2016; Pieczynska et al., 2013), and with methylene blue, which stains dead yeast cells allowing visualization of the toxin activity (Woods & Bevan, 1968).

### 2.4 Fitness in spatially structured communities

Figure S2 summarizes the fitness assays, which are described below. Mixed colonies were generated from an inoculum that contained overnight cultures of two strains. Pure colonies were generated from a single overnight culture and paired for the comparison of their initial and ending cell counts.

#### 2.4.1 Start

Two microliters of a 10 ml overnight YPD culture was added to 198 μl of water in wells of a nontreated 96‐well plate. For mixed colonies, for a 1:1 ratio, 1 μl of each strain was added; for ratios other than 1:1, 2 μl of an appropriately mixed culture was added. Three replicates were made for each strain and mix of strains; in a given 96‐well plate, only 15 wells were used, such that each experimental well was surrounded by empty wells. Cultures were then pinned onto YPD and LD‐YPD OmniTrays (Nunc 264728) using a 96‐pin multiblot replicator (V&P Scientific no. VP408FP6). For assays with toxin‐producing strains, cultures were also pinned onto low pH YPD and low pH LD‐YPD. Initial cell counts were made in one of two ways: (1) plating 100 μl of culture from the wells, and for mixed colonies either replica‐plating to appropriate antibiotic plates or viewing colonies under a fluorescence stereoscope in order to count the number of colonies of each resistance/color, or (2) imaging 10 μl of culture from the wells with a hemocytometer and differentiating strains from mixed cultures with fluorescence markers. Colonies were started with ~500–1,000 cells, as previous work has shown that starting with a low density can itself generate spatial segregation (van Gestel et al., 2014).

#### 2.4.2 Growth

For assays with nontoxin strains, YPD plates were incubated for 3 days and LD‐YPD for 5 days, both at 30°C. With toxin‐producing strains, all plates were incubated at room temperature for approximately 6–7 days (Lukša et al., 2016). For mechanical disruption, when colony growth was evident, a sterile pin was used to swirl colonies once per day. Fluorescent and/or light images were taken of each colony (Zeiss SteREO Discovery.V12 and Nikon D3200 camera) and processed in Fiji (Schindelin et al., 2012).

#### 2.4.3 End

To maximize recovery of cells for sampling, entire colonies were removed from the plates using a metal cylinder with attached rubber bulb (Figure S1). The agar plugs were suspended in either 2.5 ml water or 15% glycerol (when stored for later processing). The cylinder was sterilized via ethanol and flaming between plugs. In order to separate cells adhering to agar and/or other cells, several sterile 3.5‐mm glass beads were added to each tube and gently sonicated (UP200St sonicator with VialTweeter Sonotrode). Final cell counts were made from the processed colonies via plating a known volume or imaging with a hemocytometer, as described above.

As there are multiple sources of experimental variation in this assay, all natural strain combinations were assayed independently by two different researchers (A.H. and B.D.) using slide counts. All natural strain combinations were also assayed by plate counts to verify that cells survived colony processing. Toxin assays were performed using plate counts.

### 2.5 Competitions in liquid

Competitions were initiated with 10‐ml overnight cultures grown in the medium in which the competition would occur; 10 μl of a single strain or 10 μl of a 1:1 (by volume) mix of two strains was inoculated into 10 ml of YPD or LD. Initial counts of each culture and master mix were made using a hemocytometer. Cultures were grown at 30°C with shaking; 10 μl was serially transferred every 24 hr for either 2 or 3 cycles. After 48–72 hr from the start, final cell counts were made with a hemocytometer.

### 2.6 Statistical analysis

The data were analyzed in JMP v11.2.0 using a generalized linear model with the relative change in biofilm strain frequency as the dependent variable. Biofilm strain, treatment (medium + single vs. mixed community), assay type (plate counts vs. slide counts), and researcher were classified as discrete effects, and starting ratio was classified as a continuous effect. To meet the assumptions of normality, both starting ratio and relative change in biofilm frequency were log‐transformed before analysis.

## 3 RESULTS

To determine the fitness effect of biofilm formation during competition between unrelated genetic backgrounds, yeast strains were grown in spatially structured communities on agar plates. The experiments focused on two biofilm strains with distinct colony morphologies, which were competed against three nonbiofilm strains, and then against a toxin‐producing strain. Pairs of biofilm and nonbiofilm strains were competed against one another in both homogenous and mixed communities. Biofilm formation is induced in carbon‐limited conditions (Granek & Magwene, 2010); strain pairs were assayed with and without biofilm induction by adjusting the amount of dextrose in the medium. K2 toxin is active only in acidic conditions; strain pairs were assayed with and without toxin activity by adjusting pH.

In contrast to microbial competitions performed in liquid, spatially structured colonies do not meet the assumptions of traditional fitness calculations, specifically the requirement of a well‐mixed population (Chevin, 2010). Instead, growth is mostly limited to the front at the leading edge of the colony (Hallatschek et al., 2007). Therefore, the change in the proportion of the biofilm strain was used as a proxy for fitness. Simply by chance, a strain in a mixed colony could “win” a competition by reaching and monopolizing the edge of the colony and thus greatly increase its proportion of the population. However, by averaging over replicate colonies and assays, the effect of chance is minimized and the competitive ability of a given strain should become clear. Each of our assays included three replicates of each strain or mixed pair, and all competitions were conducted multiple times in assays performed by different researchers and with different counting methods.

### 3.1 Competitions between biofilm and nonbiofilm strains in spatially structured communities

**Figure 1:**
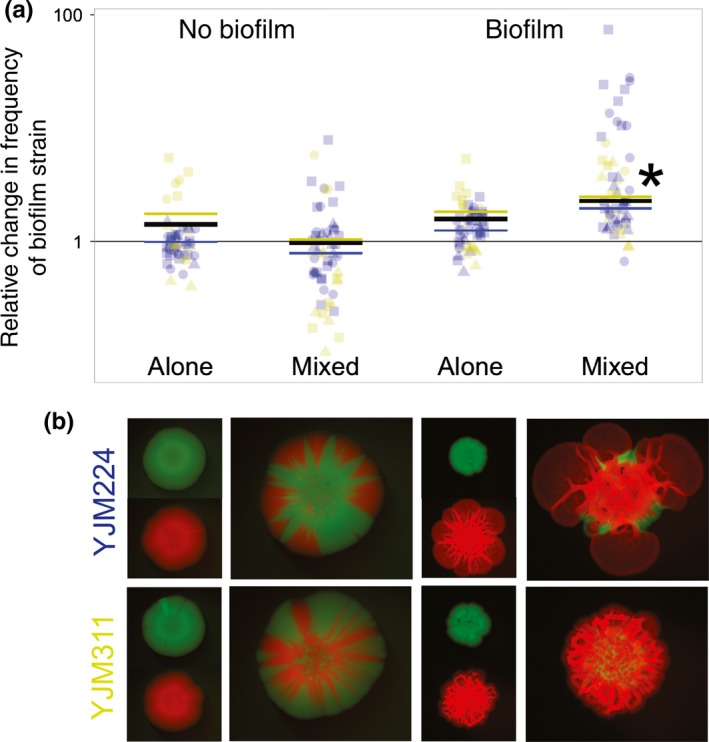
Fitness effects of biofilm formation. Biofilm‐forming strains were competed against non‐biofilm‐forming strains in pure and mixed colonies, with and without inducing biofilm formation (LD‐YPD and YPD media, respectively). For the “alone” colony treatment, colonies were paired at random and the frequency of each strain was estimated through cell counts. A total of 240 colonies were assayed. (a) Colors correspond to the biofilm‐forming strains listed in (b), YJM224 and YJM311; shapes correspond to identity of non‐biofilm‐forming strains: circle—SK1, triangle—YJM981, square—YPS681; * indicates significance at *p* < .0001. Black lines represent overall mean for a treatment; colored lines represent biofilm‐strain mean. (b) Representative images of the experimental treatments, as labeled in (a). Mixed colonies are to scale relative to one another; pure colonies are to scale relative to one another, but are scaled to half the size of the mixed colonies. Each row represents a single strain combination

Our results demonstrate a consistent and robust fitness advantage to biofilm formation in direct competition (Figure 1). In a GLM analysis of the relative change in biofilm strain frequency (Table S3), treatment was significant (*p* < .0001), while assay type (slide counts vs. plate counts) and researcher performing the experiment were not.

The LD‐mixed treatment, in which biofilms were induced in mixed‐strain communities, had the strongest positive effect on the relative change in biofilm strains (β = 0.65, *p* < .0001). Inspection of Figure 1a shows that with the exception of a single colony, the biofilm strain increased in frequency relative to the nonbiofilm strain in every mixed colony grown on LD. In contrast, in mixed communities grown on YPD, in which biofilm formation was not induced, the biofilm strain had similar or decreased fitness, with this treatment having a significant overall negative effect (β = −0.75, *p* < .0001).

Growth was also compared between homogenous, single‐strain colonies of biofilm and nonbiofilm strains. A pure colony from each strain was paired, and a starting ratio was generated with the counts of the number of cells pinned for each strain. Similarly, counts of the final number of cells in each colony were used to generate an ending ratio. In this way, the growth of the two strains could be compared, but without the strains directly competing for resources. The biofilm strains had similar fitness to nonbiofilm strains in YPD and a slight increase in LD (β = 0.15, *p* = .015). This suggests that the relative increase in the biofilm strains in mixed colonies is not simply due to faster growth in low dextrose conditions, but rather a competitive ability provided by biofilm formation.

As the starting ratio of the biofilm strain determines the ultimate possible change in the ending frequency, starting ratio was included as an effect in the model and was highly significant (β = −0.62; *p* < .0001). Through intentionally varying starting ratios, and through the inherent variation of the procedure (differing growth rates, experimental error, etc.), the starting frequency of the biofilm strain in the mixed colonies varied from 0.01 to 0.9 (Figure S4). The differences between the treatments occurred regardless of starting ratio, and in the LD‐mixed treatment, the increase in biofilm‐strain proportion was even more dramatic when starting from a low frequency.

Based on the gross morphology of the mixed colonies (Figures 1b and S3), we hypothesized that the advantage to the strains forming biofilms was due to the spatial structure of the community, specifically the ability to monopolize the leading edge of the colony. Importantly, biofilm strains appear to be able to spatially exclude nonbiofilm strains.

To test this hypothesis, a further experiment was performed based on the following logic: If the ability to increase in frequency was due to reaching and monopolizing the edge of the colony quickly, the biofilm strain's competitiveness could be hampered by mechanically disrupting the spatial structure of the community during growth (Kim et al., 2014). The original assay was performed with a third treatment that included swirling the colonies with a sterile metal sewing pin once a day. The results show only a slight, nonsignificant decrease in the fitness advantage of the biofilm strains (Figure S5; Table S4). We hypothesize that this result may be due to the frequency of mechanical disruption: 24 hr is enough time for the biofilm strain to segregate and grow before being disrupted again. However, more frequent disruption was not possible, as swirling removed small, random amounts of the colony, and regenerative growth needed to occur between disruption events.

### 3.2 Competitions between biofilm and nonbiofilm strains without spatial structure

In order to verify that the results really were due to the spatial structure and not simply due to differing growth abilities in various conditions and community compositions, the same competitions were performed with no structure at all—in well‐mixed liquid culture (Table S5, Figure S6). The results of the homogenous community treatment (strains grown alone and subsequently compared in randomly assigned pairs) recapitulated the results from the agar plates with the change in biofilm frequency around 1. In contrast, the mixed community treatment, in which two strains were grown together, showed an overall *disadvantage* to biofilm‐forming strains in direct competition. We hypothesize that this is due to the cost of producing the components of a biofilm without the associated benefits of spatial structure. These results support published findings that showed biofilm‐forming strains derived from a single genetic background grew more slowly than their smooth counterparts in liquid culture, while the diameter of the complex colonies grew more quickly than that of the smooth colonies on agar surfaces (Tan et al., 2013).

### 3.3 Competitions between K2 toxin‐producing and biofilm strains

Given the potential for yeast biofilms to gain a competitive advantage through their spatial use, and the known ability of yeast killer toxins to kill nearby sensitive cells, we sought to determine whether biofilm production protected cooperative cells or whether active toxin was effective against cells enmeshed in a biofilm. Biofilm strains and a K2 toxin strain were competed against one another in both homogenous and mixed communities, with and without inducing biofilm formation, and with and without active toxin. The toxin‐encoding virus can be lost when strains are cultured at high temperature; therefore, it was not possible to transform and fluorescently mark the toxin strains.

**Figure 2:**
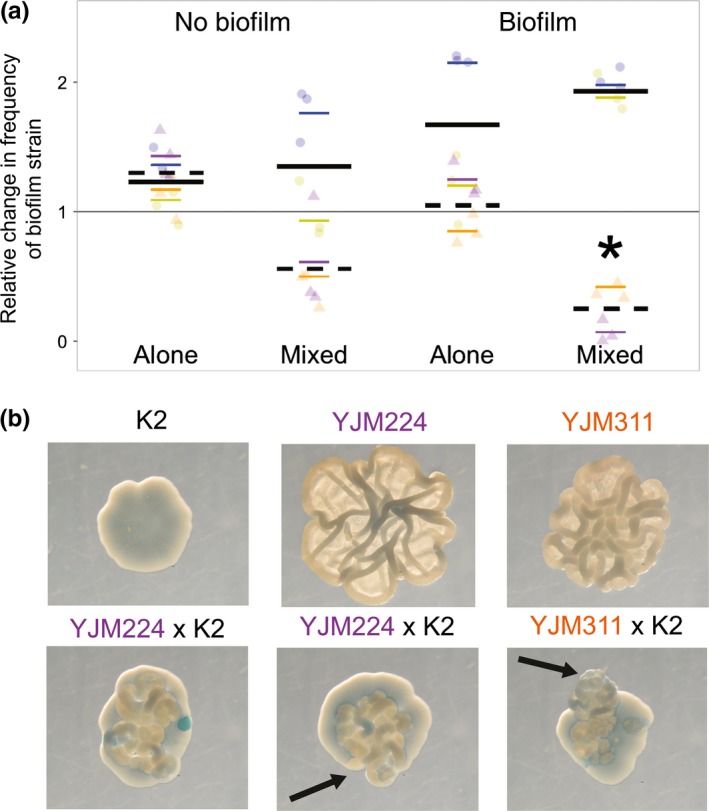
Fitness effects of biofilm formation in the presence of killer toxin. Biofilm‐forming strains were competed against toxin‐producing strains in pure and mixed colonies, with and without inducing biofilm formation, and with and without active toxin; a total of 72 colonies were assayed. (a) Yellow and blue circles correspond to fitness assays of YJM311 and YJM224, respectively, against a toxin strain, but without active toxin (as in Figure 1; toxin strain is simply another environmental isolate); solid line indicates overall mean. Orange and purple triangles refer to the same competitions, but with active toxin (low pH versions of the media); dashed line indicates overall mean; * indicates significance at *p* < .0001. (b) Representative images of single‐strain (top row) and mixed colonies (bottom row) grown on medium in which toxin is active and biofilm formation is induced (low pH, LD). Arrows indicate location of an escape of the biofilm strain at the edge of the colony. Blue dye indicates cell death

Both biofilm‐forming strains were sensitive to the toxin, as determined by halo assays (Figure S7a). In direct and indirect competition with the K2 strain, when the toxin was not active (blue and yellow in Figure 2a), the biofilm strain was more fit in nearly all treatments (Table S6). Similar to competitions with other smooth strains, the strongest fitness benefit to biofilm formation was in mixed communities (β = 0.96; *p* < .0001). In contrast, when the toxin was active (orange and purple) in mixed communities, biofilm‐forming cells were susceptible to the toxin, as indicated by the strong decrease in biofilm strain frequency (β = −1.46; *p* < .0001). Inspection of the images shows the toxin strain mostly surrounding the biofilm and dominating the edge of the colony (Figure 2b, Videos [Link], [Link], [Link]). However, in many cases, the increased spatial use by the biofilm allowed an escape at the edge of at least one section of the colony (arrows in Figure 2b).

Our results provide insight into a relationship between natural phenotypes that had not yet been explored: At least one killer toxin is effective against cells enmeshed in a biofilm, but biofilm formation may allow a sensitive strain a spatial escape.

## 4 DISCUSSION

This study investigated the fitness effects of biofilm formation in environmental isolates of the model organism, *Saccharomyces cerevisiae*; our results suggest a robust advantage in direct competition with nonbiofilm formers in spatially structured communities. In mixed colonies with biofilms induced (and without active toxin), biofilm strains consistently increased in frequency. Our results support the findings of bacterial and computational studies that show a competitive advantage associated with adhesion and spatial structure (Garcia et al., 2015; Irie et al., 2017; Kim et al., 2014; Schluter et al., 2015; Xavier & Foster, 2007), suggesting that eukaryotic microbial systems may function in a similar way.

Previous theoretical work has shown that in expanding nonbiofilm (smooth) colonies containing two genotypes, founder effects lead to sectors (as seen in Figure 1b); straight lines separating the boundaries of the sectors suggest a lack of competitive advantage, while curves suggest competition between the genotypes (Korolev et al., 2012). The mixed smooth colonies in Figures 1b and S3 suggest that strains from the different environmental backgrounds compete with one another. This supports the idea that the natural strains are in competition for resources; this competition is likely also occurring in the mixed biofilm colonies. Thus, the dominance of the biofilm‐forming strains could be due to a competitive function of the biofilms.

In contrast to the mixed‐strain colonies, our data showed either no, or a slight, fitness advantage to biofilm formation in indirect competition between single‐strain colonies. These results are in agreement with Regenberg et al. (2016), who showed that the fitness benefit of yeast biofilm formation increased as the viscosity of the medium decreased; at 2% agar in their study, the concentration used here, the dry biomass of biofilm and nonbiofilm colonies was not different. In these laboratory studies, the choice of agar concentration is somewhat arbitrary, as it is impossible to recapitulate unknown natural conditions. While *S. cerevisiae* has been isolated in numerous ecological niches, usually associated with fruits or man‐made environments, it is unclear what habitat it evolved in and was historically adapted to (Goddard & Greig, 2015). Regardless, it is interesting that even in conditions that do not provide a fitness advantage to biofilm formation in indirect competition, biofilms still provide a competitive advantage in direct competition.

Next, this study investigated a less‐understood interaction between two social phenotypes: biofilm formation and toxin production. Our results suggest that in this eukaryotic system, the toxin was effective at containing the growth of the biofilm strain. However, even in the presence of toxin, both biofilm strains were able to reach the leading edge of the colony and grow outward, potentially serving as a spatial escape. While the assay was performed in an artificial laboratory setting, the results suggest that increased use of space by yeast biofilms may not only provide an escape from competition for nutrients, but may also provide an escape from warfare phenotypes. It is interesting to note that the toxin and Flo11p, the cellular adhesin responsible for cell–cell attachment, are both most effective in acidic conditions (Kraushaar et al., 2015; Lukša et al., 2016); we thus speculate that yeast biofilms and toxins could interact in the natural environment.

Biofilms represent the earliest form of multicellular structures in the evolution of life and are currently found in numerous natural and man‐made environments—from water filtration systems, to dental surfaces, to medical implants—and can pose a serious threat to human health. Thus, understanding biofilms may not only lead to insights into the evolution of early microbial communities, but may have practical implications. Our study demonstrates that biofilms provide a competitive fitness benefit to a eukaryotic species, as they do to bacterial species, and suggests that eukaryotic microbes may face similar selective pressures. Thus eukaryotes may meet the assumptions of much of the in silico microbial social evolution research focused on spatially explicit communities. Furthermore, we show that the premier biomedical model yeast, *S. cerevisiae*, may be a powerful system to investigate questions surrounding social evolution in eukaryotic biofilms, an area of research that has received little attention.

## CONFLICT OF INTEREST

The authors declare no conflict of interests.

## AUTHOR CONTRIBUTIONS

BMD and ARH conducted colony fitness assays; BMD conducted toxin assays; BAL conducted liquid fitness assays; HAM conceived of the project, generated strains, and conducted statistical analysis. HAM and BMD wrote the manuscript.

## DATA ACCESSIBILITY

All data are archived at https://doi.org/10.5061/dryad.3b21m43.
