# Supplementary material for: Biofilm formation and toxin production provide a fitness advantage in mixed colonies of environmental yeast isolates
Source: Ecol Evol. 2018 Apr 27;8(11):5541–50. doi: 10.1002/ece3.4082 (PMC6010761; doi:10.1002/ece3.4082)
Supplement: Supplementary file 1 [file ECE3-8-5541-s001.pdf]

Electronic Supplementary Material

"Biofilm formation and toxin production provide a fitness advantage in mixed colonies of environmental yeast isolates"

Bernadette M. Deschaine, Angela R. Heysel, B. Adam Lenhart, Helen A. Murphy

This file contains Tables S1-S6 and Figures S1-S7.

Videos:
VS1: Biofilm Formation of YJM224
Time lapse video over 96 hours; images taken every 6 hours. Left panel- bright field images; right panel- red fluorescent.

VS2: Colony Formation of K2
Time lapse video over 96 hours; images taken every 6 hours. Left panel- bright field images; right panel- red fluorescent. K2 was not marked with fluorescence.

VS3- Mixed Colony Formation of YJM224 x K2
Time lapse video over 96 hours; images taken every 6 hours. Left panel- bright field images; right panel- red fluorescent. YJM224 was marked with mCherry; K2 was not marked with fluorescence. Blue dye indicates death. By the end, the biofilm is surrounded by the toxin strain, with the exception of the top left corner.

Background/ Isolate	Source	Person/ Collection	Citation	Murphy Lab #	mCherry-KanMX	mCherry-HphMX	GFP-KanMX	GFP-NatMX	GFP-HphMX
YJM311	Clinical, bile tube, San Francisco, USA	Paul Magwene (PMY144)	McCusker *et al.*, 1994	HMY3	HMY7				
YJM224	Distillery Yeast	Paul Magwene (PMY133)		HMY2	HMY12	HMY247	HMY9	HMY246	
YJM981	Clinical, vaginal, Bergamo, Italy	Sanger Collection	Liti *et al.*, 2009	HMY157			HMY234		
SK1	Lab strain, soil, USA	Sanger Collection	Liti *et al.*, 2009	HMY169			HMY210		HMY224
YPS681	Woodland, oak tree, Buck Hill Falls, PA, USA	Paul Sniegowski	Sniegowski *et al.*, 2002	HMY270			HMY10	HMY211	HMY230
L-1528	Vineyard, Cauquenes, Chile, fermentation must from Cabernet	Sanger Collection	Liti *et al.*, 2009	HMY160			HMY222		HMY252

Toxin Strain				
29-06	K2	Dominika Wloch-Salamon	Pieczynska *et al.*, 2013	HMY315

Table S-1: Strains

**PGK1-YRC-for**	GGTAAGGAATTGCCAGGTGTTGCTTTCTTATCCGAAAAGAAAGGTCGACGGATCCCCGGG	
**PGK1-YRC-rev**	GAAAAGAAAAAAATTGATCTATCGATTTCAATTCAATTCAATATCGATGAATTCGAGCTCG	
	40 bp upstream and downstream of the *PGK1* stop codon plus homology to the pFA6a plasmid (GFP-KanMX cassette) and pBS34 (mCherry-KanMX). Annealing temperature should be based on homology to the plasmid (~45°C).	
**MX-for**	CGTACGCTGCAGGTCGAC	
**MX-rev**	ATCGATGAATTCGAGCTCG	
	Universal MX primers	

Table S-2: Primers

Source	DF	Likelihood Ratio	p-value
Starting Ratio	1	147.397	<0.0001*
Medium	3	135.058	<0.0001*
Biofilm Strain	1	1.177	0.2789
Medium* Biofilm Strain	3	4.802	0.1869
Assay Type	1	0.307	0.5797
Researcher	1	0.012	0.9121

Significant Effects	Estimate	Standard Error	p-value
Intercept	-0.4862	0.061	<0.0001*
Starting Ratio	-0.6204	0.043	<0.0001*
LD Mixed	0.6502	0.065	<0.0001*
LD Alone	0.1594	0.065	0.0151*
YPD Alone	-0.0547	0.065	0.4020
YPD Mixed	-0.7549	0.065	<0.0001*

Table S-3: Generalized linear model for relative change in biofilm frequency after colony growth with non-toxin strains. Relative change in biofilm frequency and starting ratio were log-transformed before analysis.

Effect	DF	Likelihood ratio	p-value
Starting Ratio	1	94.06	<0.0001*
Medium	3	105.78	<0.0001*
Biofilm Strain	1	0.125	0.7232
Disruption Treatment	1	0.102	0.7495
Medium* Biofilm Strain	3	4.62	0.2016
Medium*Disruption	3	3.02	0.3885
Disruption*Biofilm Strain	1	0.531	0.4662
Medium* Disruption*Biofilm Strain	3	0.818	0.8452

Table S-4: Generalized linear model for interactions between biofilm and non-toxin strains with and without mechanical disruption of the colonies. Relative change in biofilm frequency and starting ratio were log-transformed before analysis.

Effect	DF	Likelihood-ratio	p-value
Starting Ratio	1	10.18	0.0014*
Medium	3	27.91	<0.0001*
Biofilm Strain	1	0.196	0.6583
Medium* Biofilm Strain	3	15.94	0.0012*

Effects of Medium	Estimate	Standard Error	p-value
Starting Ratio	1.91	0.59	0.0014*
LD Mixed	-0.77	0.22	0.0006*
LD Alone	0.73	0.31	0.0176*
YPD Mixed	-0.92	0.23	<.0001*
YPD Alone	0.96	0.31	0.0020

Table S-5: Generalized linear model for interactions between biofilm strains in liquid media. Relative change in biofilm frequency and starting ratio were log-transformed before analysis.

Effect	DF	Likelihood-ratio	p-value
Starting Ratio	1	6.30	0.0121*
Medium	7	130.67	<0.0001*
Biofilm Strain	1	0.0651	0.7986
Medium* Biofilm Strain	7	72.49	<0.0001*

Effects of Medium	Estimate	Standard Error	p-value
LD Mixed	0.955	0.109	<.0001*
LD Alone	0.324	0.109	0.0044*
YPD Mixed	0.536	0.109	<.0001*
YPD Alone	0.050	0.109	0.6441
LD Mixed, low pH	-1.458	0.109	<.0001*
LD Alone, low pH	-0.114	0.109	0.2958
YPD Mixed, low pH	-0.402	0.109	0.0005*
YPD Alone, low pH	0.109	0.109	0.3138

Table S-6: Generalized linear model for interactions between biofilm strains and a K2 toxin strain. Relative change in biofilm frequency and starting ratio were log-transformed before analysis.

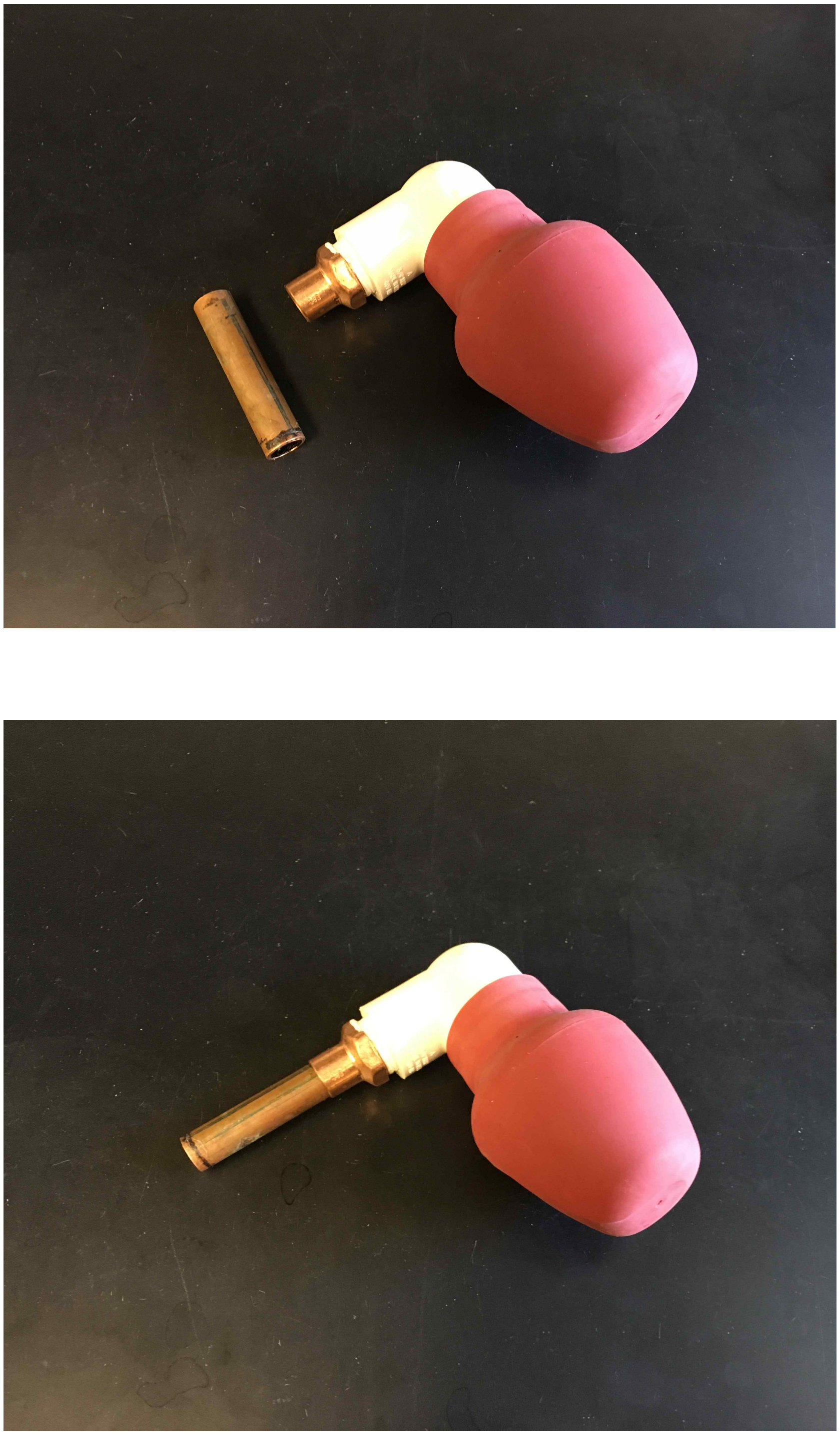

Figure S1: Picture of tool used to remove agar plugs with embedded colonies.

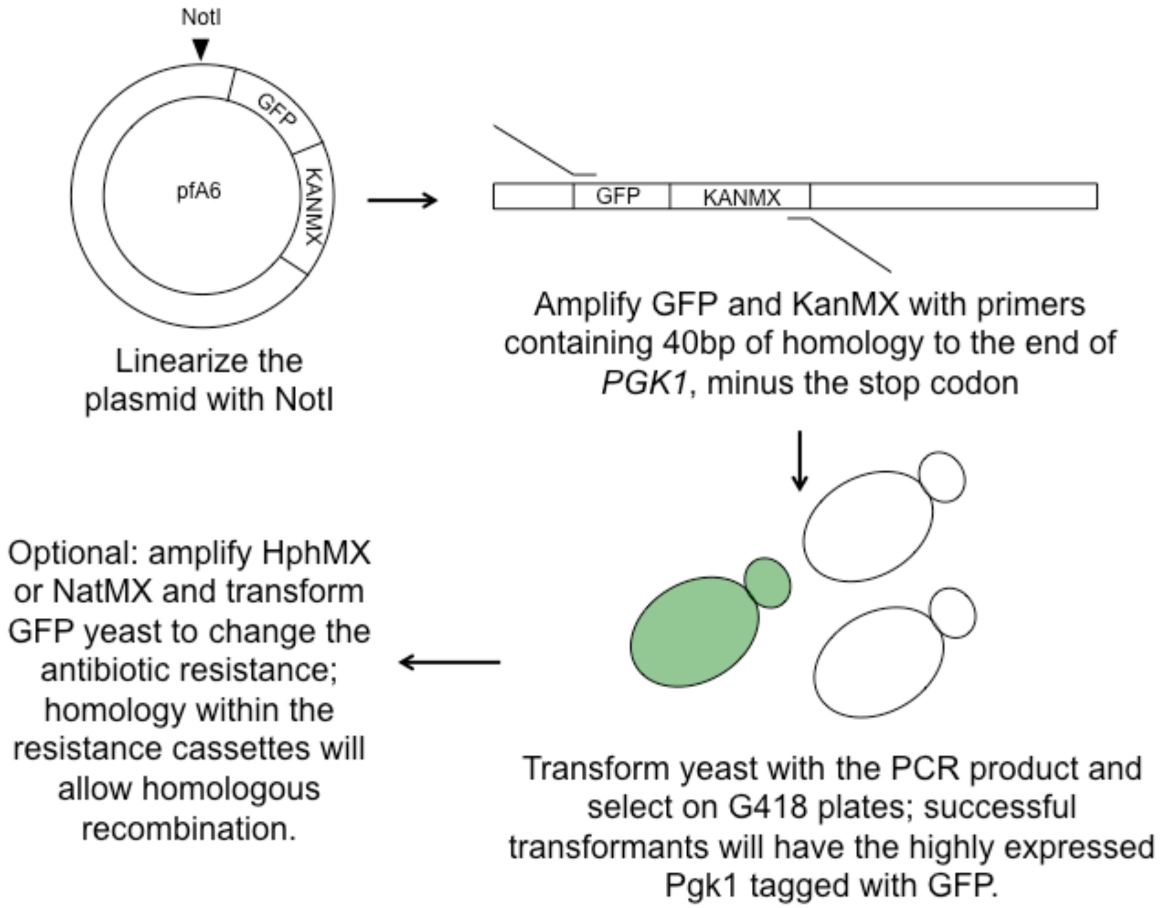

Figure S2a. Schematic of the methods: Engineering yeast strains. Either pFA6-GFP-KanMX (Longtine et al. 1998) or pBS34 (Hailey et al. 2002), which contains mCherry-KanMX, can be used to target any gene.

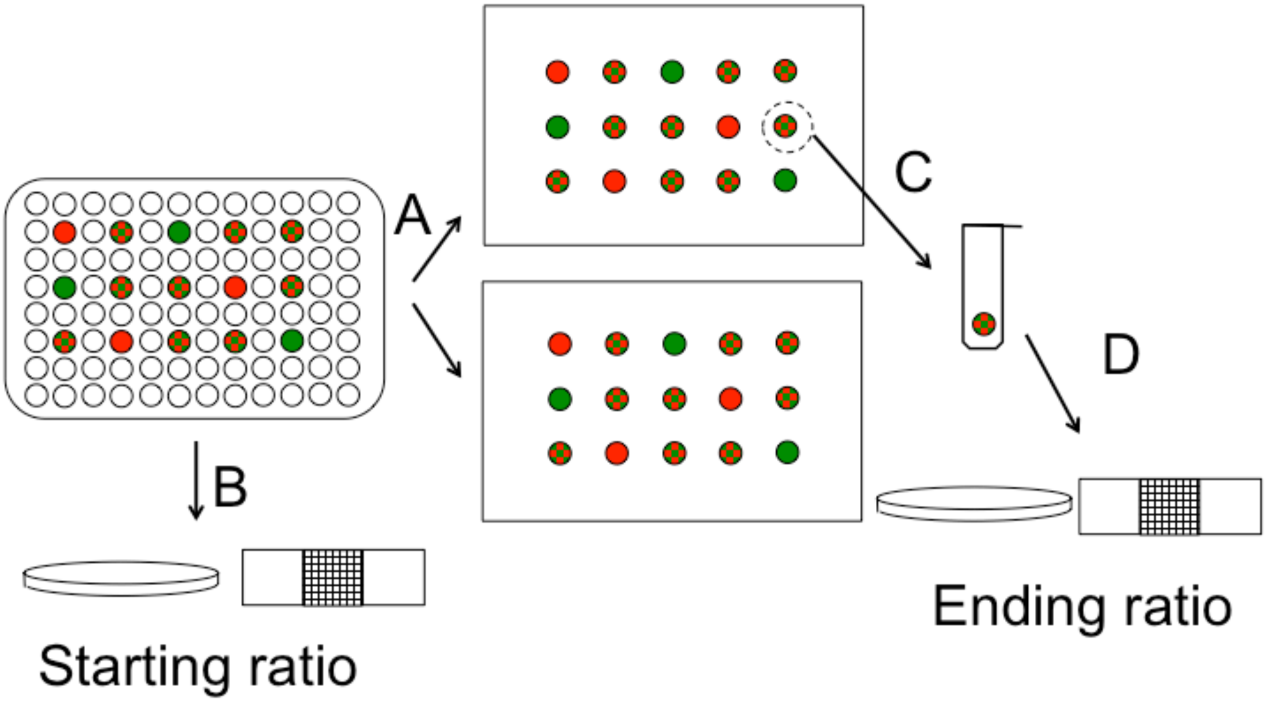

Figure S2b. Schematic of the methods: Fitness assay. (A) Overnight cultures are diluted, mixed when appropriate, and used to fill replicate wells in a 96-well plate. The cultures are pinned to multiple agar plates containing medium that controls biofilm formation and toxin production. (B) A sample of each populated well is either: (1) plated, then replica-plated to antibiotic plates, or (2) visualized using a hemocytometer and fluorescent microscope; fluorescence and antibiotic markers allow each strain/colony to be differentiated. (C) After multiple days of growth, each colony is removed using the tool in Figure S1, and resuspended in water through sonication with glass beads. (D) A sample of the colony suspension is either plated or visualized as in B.

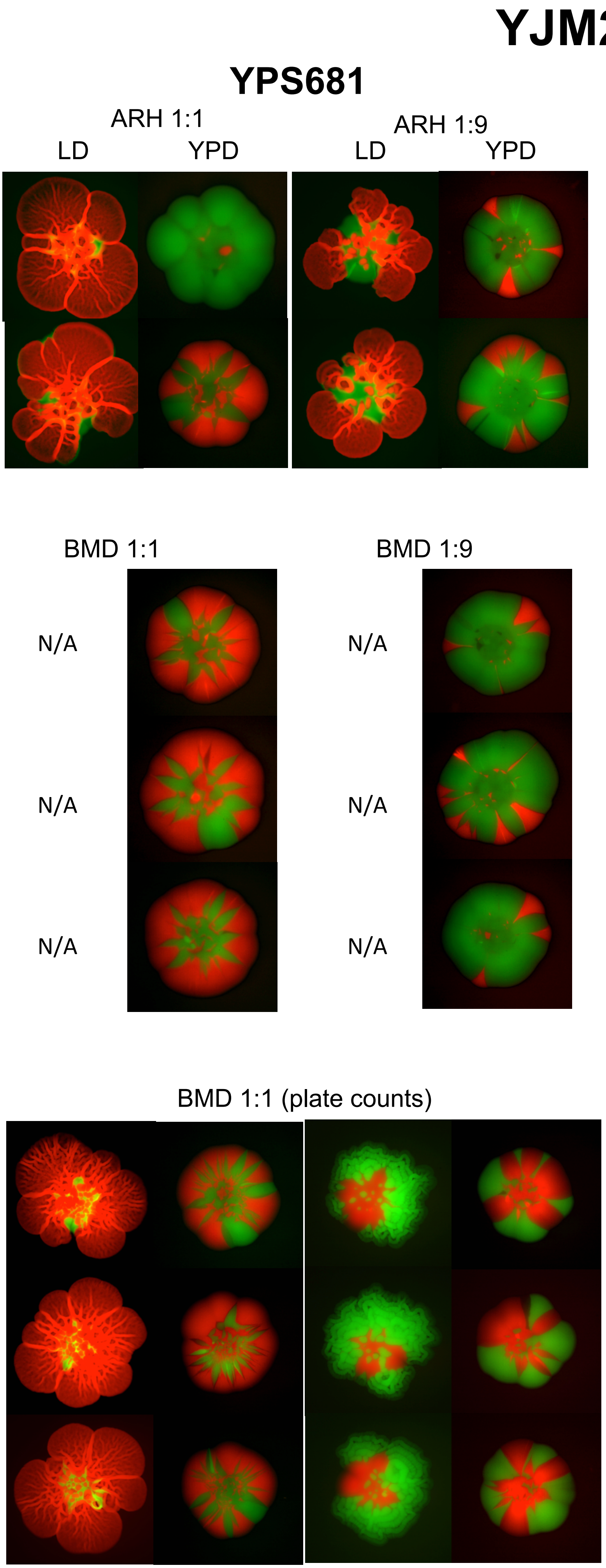

Figure S3: Mixed-community yeast colonies. Biofilm formation/complex colony morphology was induced on low dextrose (LD) YPD; non-biofilm/smooth colony formation occurred on normal YPD. Each culture in a multi-well plate was pinned onto an LD and YPD plate. Images next to each other represent matching pairs of colonies (biofilm and non-biofilm). In general, there were 3 replicate cultures for most treatments and assays.

ARH and BMD refer to the researcher who performed the assay.

The ratio refers to the starting ratio (by volume) of the two strains.

N/A indicates issues with the image file, not issues with the actual colony or resulting count data.

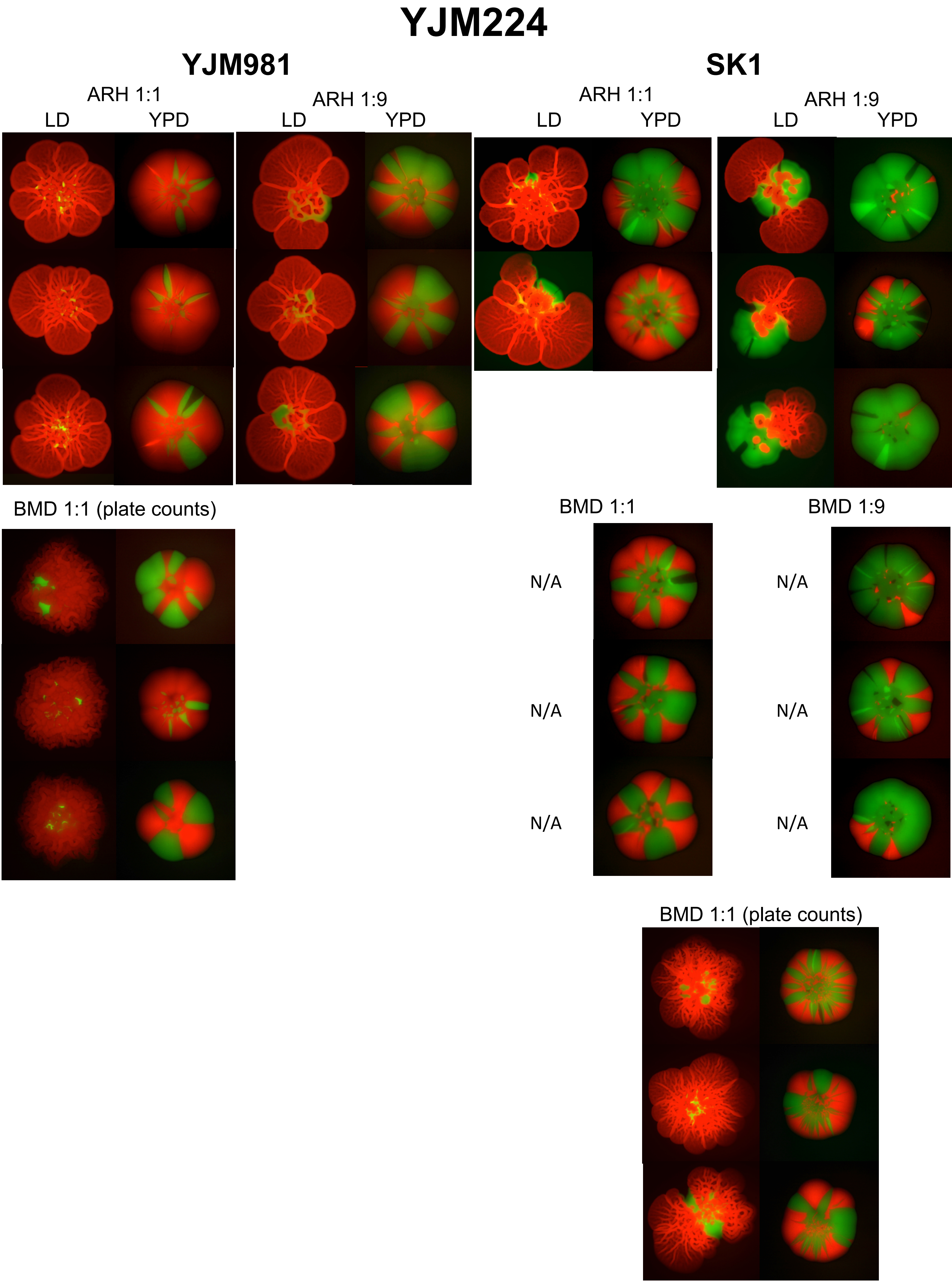
YJM224
YJM981
SK1
ARH 1:1
ARH 1:9
LD
YPD
BMD 1:1 (plate counts)
BMD 1:1
BMD 1:9
N/A

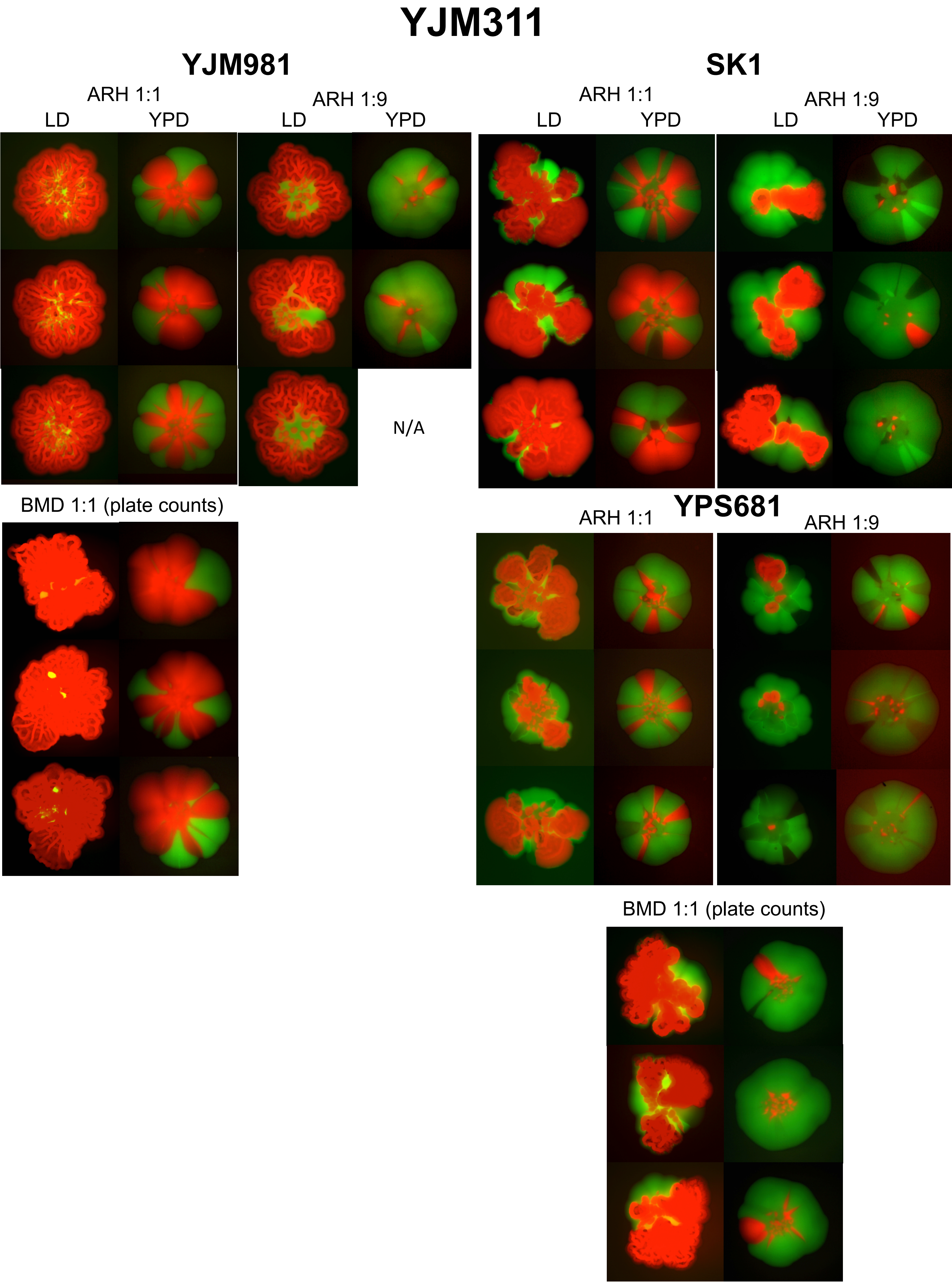
YJM311
YJM981
ARH 1:1
LD
YPD
ARH 1:9
LD
YPD
N/A
BMD 1:1 (plate counts)
SK1
ARH 1:1
LD
YPD
ARH 1:9
LD
YPD
YPS681
ARH 1:1
ARH 1:9
BMD 1:1 (plate counts)

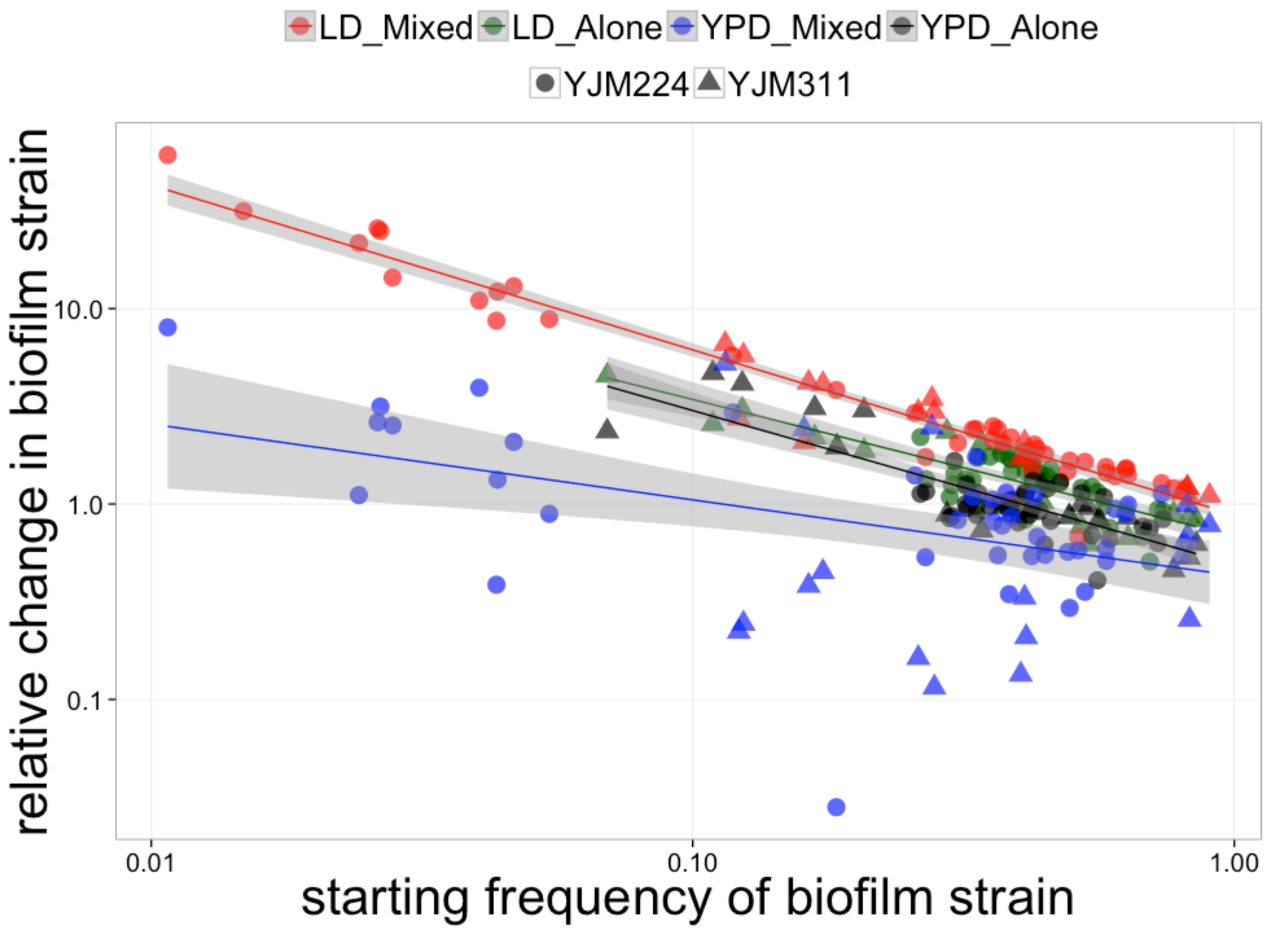

Figure S4: Competitions between environmental isolates over a range of starting biofilm strain frequencies. Circles and triangles correspond to biofilm-forming strains YJM224 and YJM311, respectively. Colors correspond to treatment, where LD induces biofilm formation and YPD does not. Each data point corresponds to a single competition; data are plotted on a log-log scale. Lines with 95% confidence intervals were calculated in R with the lm function.

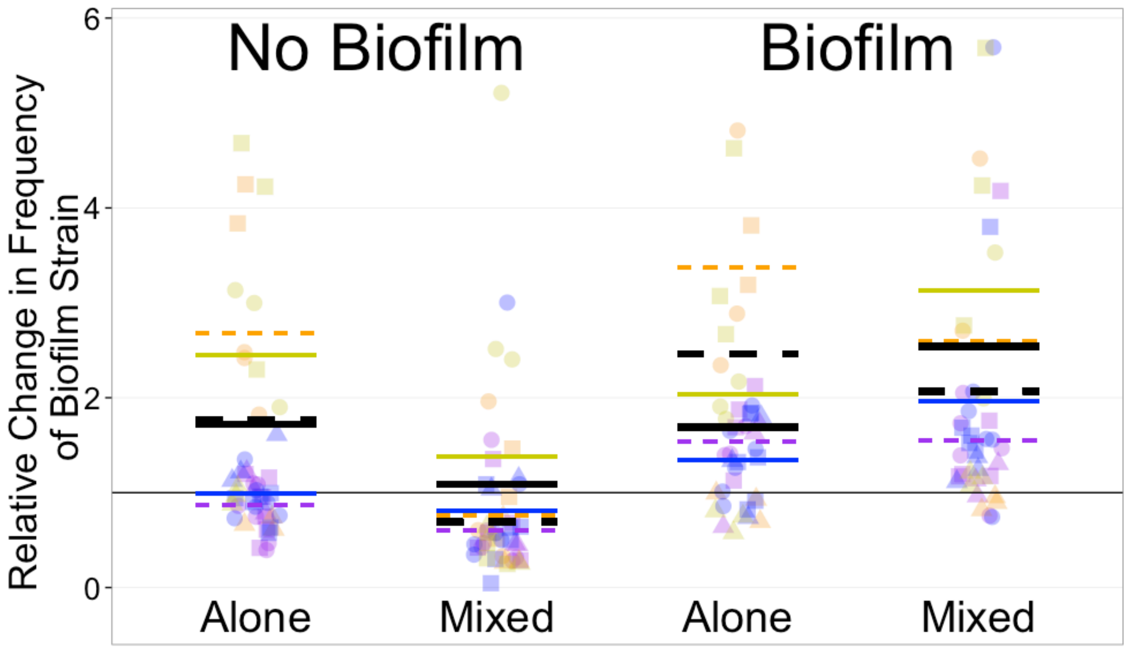

Figure S5: Competitions between environmental isolates with mechanical disruption of the communities. Blue and yellow correspond to biofilm-forming strains YJM224 and YJM311 without disruption; purple and orange correspond to YJM224 and YJM311 with disruption; shapes correspond to identity of non-biofilm forming strains: circle- SK1, triangle- YJM981, square- YPS681. Solid black lines represent overall mean for non-disruption treatments; dashed black lines correspond to overall mean for disruption treatments; colored lines represent biofilm strain mean.

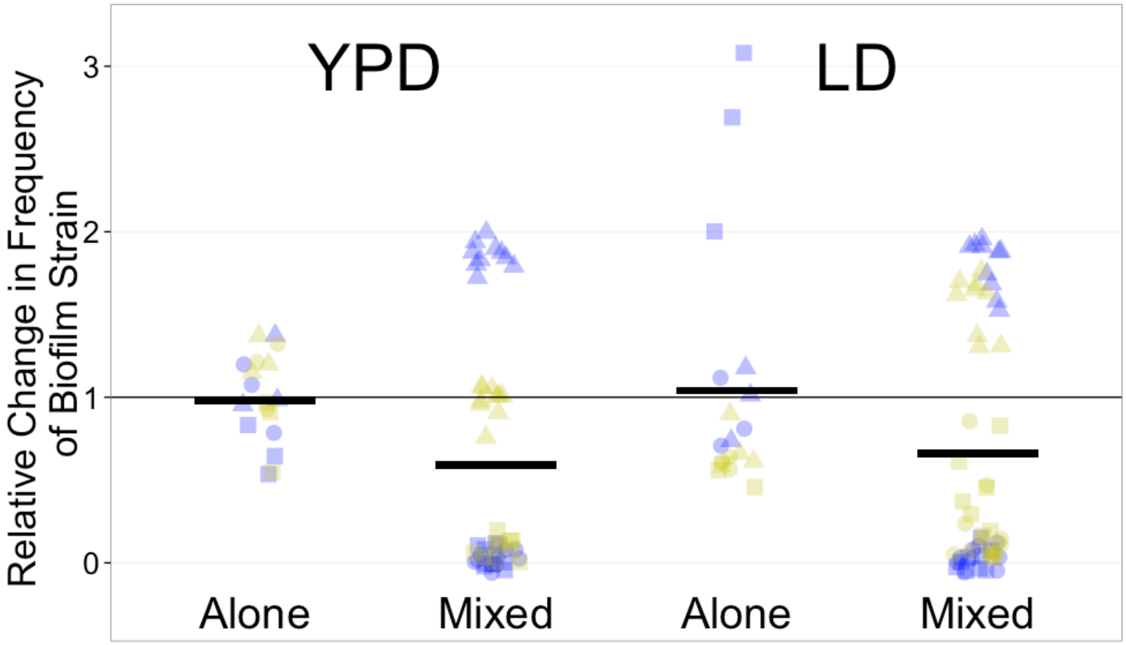

Figure S6: Competitions in liquid. Blue and yellow correspond to biofilm-forming strains YJM224 and YJM311, respectively; shapes correspond to identity of non-biofilm forming strains: circle- SK1, triangle- YJM981, square- YPS681. Black lines represent overall mean for treatments. In competitions between randomly paired single-strain cultures, the results are similar to spatially structured communities, with the average change in biofilm frequency around 1. The one exception is YPS681, which had a slower growth rate in low dextrose conditions compared to YJM224. In mixed liquid cultures, the results suggest that the biofilm strains are overall less competitive than when in spatially structured communities; however, YJM981 appears to be a poor competitor in liquid.

A

Halo Assays

YJM311 **YJM224**

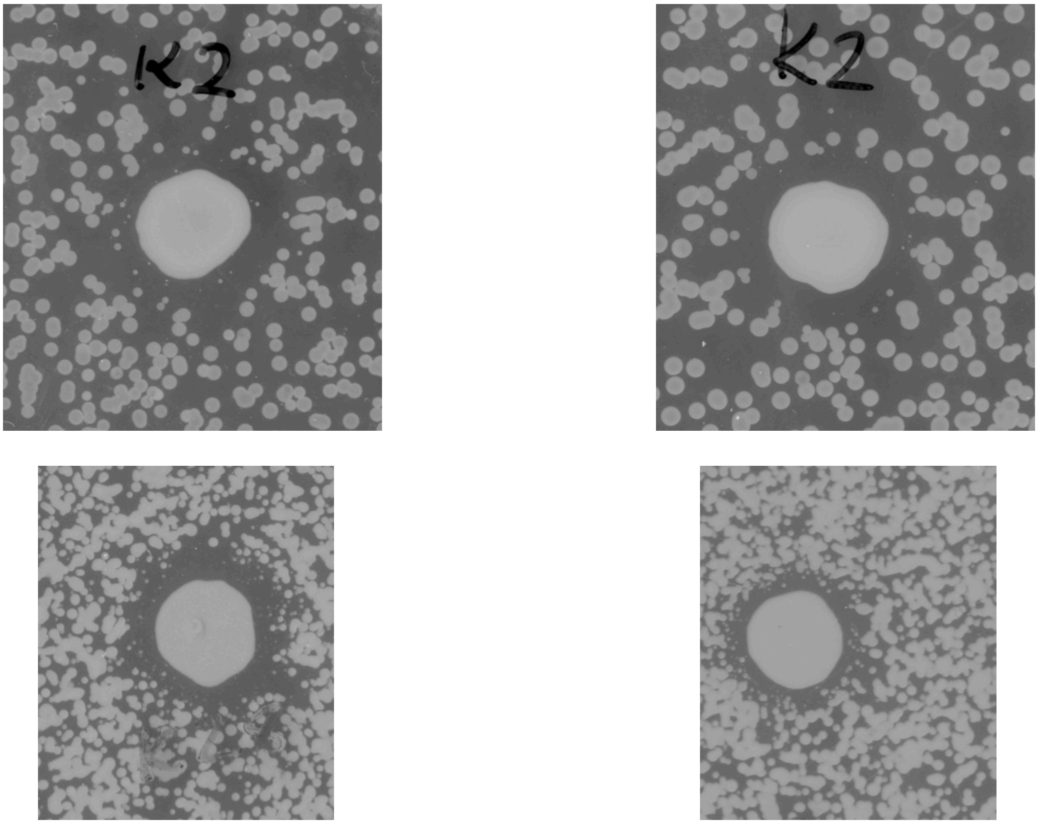

Figure S7: (A) Halo Assays. Overnight cultures of YJM311 and YJM224 were plated in two different densities; 20 ul of overnight culture of K2 was put in a droplet on the plates. The haloes are zones of death where the sensitive strains could not grow. **(B) Mixed community yeast colonies with toxin strains.** Biofilm formation/ complex colony morphology was induced on low dextrose (LD) YPD; non-biofilm/ smooth colony formation occurred on normal YPD; toxin was active in low pH. Each culture in a multi-well plate was pinned onto LD 2% agar, YPD 2% agar, LD low pH 1.5% agar, and YPD low pH 1.5% agar plates. Images next to each other represent colonies from the same initial well.

N/A indicates issues with the image file, not issues with the actual colony or resulting count data.

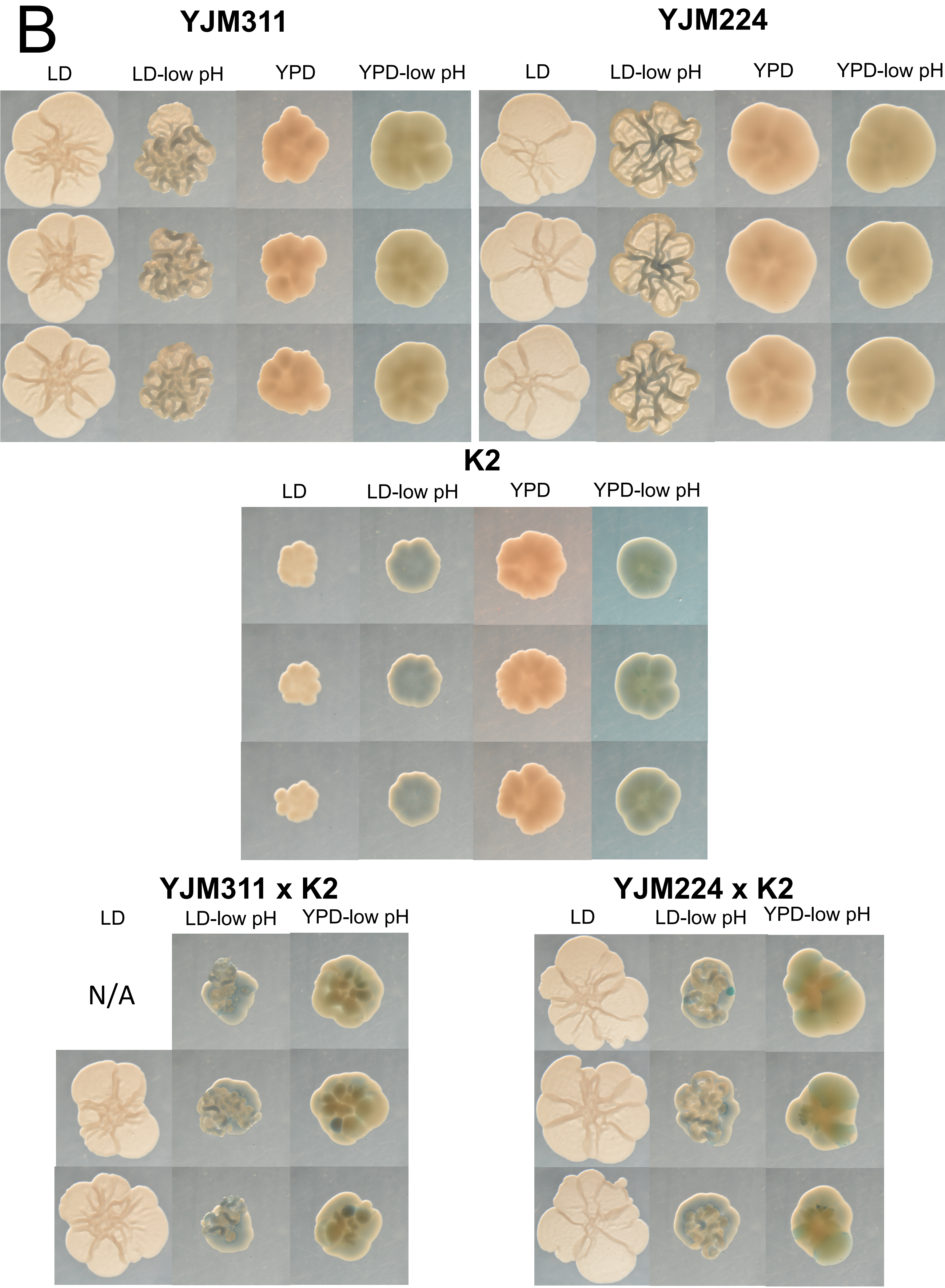

B
YJM311
YJM224
LD
LD-low pH
YPD
YPD-low pH
K2
YJM311 x K2
YJM224 x K2
N/A
